# Alzheimer-Compound Identification Based on Data Fusion and forgeNet_SVM

**DOI:** 10.3389/fnagi.2022.931729

**Published:** 2022-07-25

**Authors:** Bin Yang, Wenzheng Bao, Shichai Hong

**Affiliations:** ^1^School of Information Science and Engineering, Zaozhuang University, Zaozhuang, China; ^2^School of Information and Electrical Engineering, Xuzhou University of Technology, Xuzhou, China; ^3^Department of Vascular Surgery, Zhongshan Hospital (Xiamen), Fudan University, Xiamen, China

**Keywords:** virtual screening, network pharmacology, Alzheimer, data fusion, feature selection, machine learning

## Abstract

Rapid screening and identification of potential candidate compounds are very important to understand the mechanism of drugs for the treatment of Alzheimer's disease (AD) and greatly promote the development of new drugs. In order to greatly improve the success rate of screening and reduce the cost and workload of research and development, this study proposes a novel Alzheimer-related compound identification algorithm namely forgeNet_SVM. First, Alzheimer related and unrelated compounds are collected using the data mining method from the literature databases. Three molecular descriptors (ECFP6, MACCS, and RDKit) are utilized to obtain the feature sets of compounds, which are fused into the all_feature set. The all_feature set is input to forgeNet_SVM, in which forgeNet is utilized to provide the importance of each feature and select the important features for feature extraction. The selected features are input to support vector machines (SVM) algorithm to identify the new compounds in Traditional Chinese Medicine (TCM) prescription. The experiment results show that the selected feature set performs better than the all_feature set and three single feature sets (ECFP6, MACCS, and RDKit). The performances of TPR, FPR, Precision, Specificity, F1, and AUC reveal that forgeNet_SVM could identify more accurately Alzheimer-related compounds than other classical classifiers.

## Introduction

Alzheimer's disease (AD) is the most common type of senile dementia, which is a frequently occurring disease of the elderly (Romanelli et al., 1990; Morán et al., 1992; Wang et al., 2014). Its main clinical manifestations are the decline of cognitive function, mental symptoms and behavior disorders, and the decline of daily living ability (Almeida and Crocco, 2000; Daulatzai, 2014; Zhao et al., 2016; Gong et al., 2017). It poses a great threat to the health and quality of life of the elderly and brings a heavy economic burden to society (Rice et al., 1993; Rothstein et al., 1996; Hu, 2006; Wang, 2014). The main reason for the onset of AD is the central nervous system disease in the brain, which causes a series of mental diseases such as learning impairment, memory impairment, and speech impairment (Ogomori et al., 1989; Hao et al., 2013). Family inheritance, physical diseases, and head trauma can cause the onset of this disease (Heyman, 1994; Mehta et al., 1999). However, in the process of studying the pathogenesis of AD, there are some problems such as unclear pathogenesis, difficult early diagnosis, and no preventable and curable drugs. Therefore, the diagnosis and treatment of AD have been a difficult problem for medical researchers in recent decades.

Alzheimer is a complex disease with multiple factors. At present, the main drugs for the treatment of AD in clinics are acetyl cholinesterase inhibitors, glutamate receptor inhibitors, etc. (Liston et al., 2004; Dong et al., 2005; Sugimoto, 2006). These drugs can alleviate the symptoms caused by the decline of cognitive function, but cannot fundamentally eliminate the pathogeny. Network pharmacology is based on multi-disciplinary knowledge such as system biology, multi pharmacology, bioinformatics, computer technology, and network analysis (Berger and Iyengar, 2009; Chen et al., 2012; Yuan et al., 2019; Li et al., 2020). It systematically studies the drug-target-pathway-disease interaction network and discusses the multi-component, multi-target, and multi-channel pharmacological mechanism of traditional Chinese medicine (TCM) (Li et al., 2014; Xiong et al., 2018; Jiang et al., 2020; Gao et al., 2021). It plays a very important role in exploring treatment approaches and clarifying drug efficacy, especially in finding the effective components of drugs, which is highly consistent with the holistic view emphasized by the theory of traditional Chinese medicine. In recent years, a variety of traditional Chinese medicine prescriptions have been proposed to improve AD by network pharmacology from point of view of multi-component, multi-target, and multi-channel (Sun et al., 2017; An et al., 2020; Wang et al., 2020; Huang et al., 2021). Pang et al. analyzed 25 targets and 13 TCM prescriptions for the treatment of AD and selected 7 representative Chinese medicines (Pang et al., 2016). Naive Bayesian and recursive partitioning was utilized to predict the targets contributing to the chemical components of traditional Chinese medicine in order to construct a compound-target-disease network and explain the synergistic mechanism of multiple effective components of TCM prescriptions. Tao et al. analyzed the compounds of Paeoniae Rubra Radix and Phellodendri Cortex, and the Alzheimer-related targets to reveal the mechanism of these two medicinal materials for intervening AD (Tao et al., 2015). Wang et al. analyzed the main active components of Liuwei Dihuang Decoction and the main action targets of active components and carried out the GO and pathway analyses to give the multi-component, multi-channel and multi-target mechanism of Liuwei Dihuang Decoction in the treatment of AD (Wang et al., 2021). Jiang and Wang utilized network pharmacology to analyze the mechanism of Bajitian for treating AD and obtain that this drug could play an anti-pharmacological role in many aspects, such as neurotransmitter, regulation and regulation of ion channels (Jiang and Wang, 2021).

In network pharmacology, screening the main active compounds of prescriptions is an essential step. In past studies, this step is processed mainly by manually searching public databases. In this study, a novel machine learning method, namely forgeNet_SVM is proposed to identify Alzheimer-related active compounds. The data mining method is utilized to collect Alzheimer-related compounds from the literature. Three molecular descriptors (ECFP6, MACCS, and RDKit) are utilized to obtain the feature sets of compounds respectively, which are fused into an all_feature set. The all_feature set is input to the forgeNet_SVM, in which forgeNet is utilized to give the importance of each feature and select the important features for feature extraction. The selected features are input to support vector machines (SVM) algorithm to identify the new AD-related compounds in TCM prescription.

## Methods

### forgeNet

**Figure 1:**
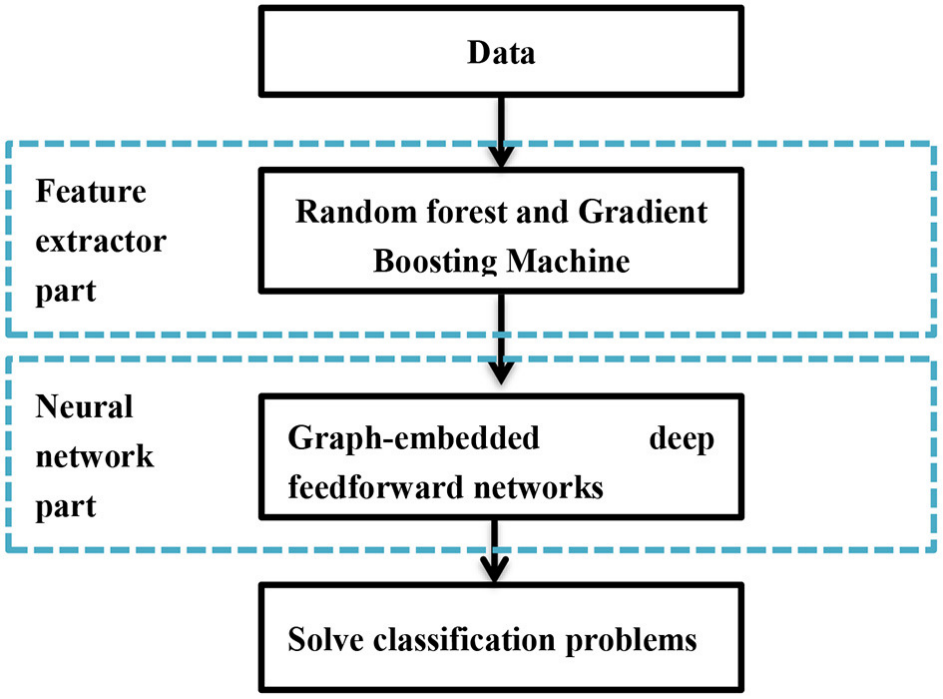
The flowchart of forgeNet algorithm.

Forest graph-embedded deep feed forward network (forgeNet) is based on ensemble method and deep learning, which has been utilized for gene regulatory network inference and biology data classification (Kong and Yu, 2020; Yang, 2021). Figure 1 shows the framework of forgeNet, in which the development of feature graph and classification of deep learning model are contained. Compared to classical deep learning models, forgeNet could solve the dimension imbalance of biomedical data and is more robust (Kong and Yu, 2020).

#### Development of Feature Graph

With the dimension-imbalance data, the important features of the data are selected for feature extraction. Thus, forgeNet utilizes forest ξ, which includes *p* decision trees (DTs). According to the training dataset with the classification labels, ξ is fitted and *p*DTs could be created (ξ(θ) = {*T*_1_(θ_1_), *T*_2_(θ_2_), …, *T*_*p*_(θ_*p*_)}, θ_*i*_is the coefficient). If a binary tree is considered a special case of a directed graph, the graph set could be obtained as follows.


(1) $$\begin{array}{l} \text{Φ}=\left\{G_{1}\left(V_{1},\;E_{1}\right),\;\ldots ,G_{i}\left(V_{i},\;E_{i}\right),\;\ldots ,\;G_{N}\left(V_{p},\;E_{p}\right)\right\}. \end{array}$$

Where *V*_*i*_ and *E*_*i*_denote vertex and edge sets of *G*_*i*_, respectively.

In order to combine the directed graph set Φ, we can obtain the final aggregated graph as follows.


(2) $$\begin{array}{l} G=\overset{p}{\underset{i=1}{⋃}}G_{i}. \end{array}$$

#### Classification of Deep Learning Model

According to the feature graph obtained from the previous step, graph-embedded deep feed forward networks (GEDFN) are utilized to train in order to obtain the optimal model, which is utilized to provide the classification results of the unknown data (Yang, 2021). Every layer of GEDFN is given as followed.


(3) $$\begin{array}{l} \begin{array}{l} Z_{1}=\sigma \left(X\left(W_{in}\text{Θ}G\right)+b_{in}\right),\\ \ldots \\ Z_{k+1}=\sigma \left(Z_{k}W_{k}+b_{k}\right),\\ \ldots \\ Z_{out}=\sigma \left(Z_{l}W_{l}+b_{l}\right),\\ y=soft\text{ max}\left(Z_{out}W_{out}+b_{out}\right). \end{array} \end{array}$$

Where *X* represents input vector, *Z*_*k*_ denotes the *k* − *th* hidden layer, Θ is Hadamard product, *W*_*k*_ and *b*_*k*_ are the weight and bias of the *k* − *th* hidden layer, respectively.

forgeNet also gives a feature importance evaluate mechanism, which is based on Graph Connection Weights (GCW) method (Kong and Yu, 2018). The score of *i* − *th*feature is defined as follows.


(4) $$\begin{array}{r} c_{i}=\overset{n}{\underset{a=1}{\sum }}|W_{ia}^{\left(in\right)}T\left(A_{ia}=1\right)|+\overset{n}{\underset{b=1}{\sum }}|W_{bi}^{\left(in\right)}T\left(A_{bi}=1\right)|\\ +\overset{b_{1}}{\underset{c=1}{\sum }}|W_{ic}^{\left(1\right)}T\left(A_{ia}=1\right)|. \end{array}$$

Where *n* is the number of features in the dataset, *W*^(*in*)^ represent the weights between the input layer and the first hidden layer, and *W*^(1)^ represent the weights between the first hidden layer and the second hidden layer. After forgeNet is trained, the importance scores for all the features could be computed with the trained weights.

### Support Vector Machine

Support vector machine (SVM) is one of the most classical machine learning algorithms, which was proposed in the year 1995 (Cortes and Vapnik, 1995). SVM is suitable for the classification problems of small-medium samples, nonlinear, and high-dimensional pattern recognition. The basic principle of SVM is to find an optimal classification surface (Hyperplane), which can not only separate the samples without errors but also maximize the margin, based on the most classification surface in the case of linear separability (Suykens and Vandewalle, 1999; Saunders et al., 2002). Therefore, the learning process of SVM is an optimization problem.

The training dataset contains *N* sample points {(*x*_1_, *y*_1_), …, (*x*_*N*_, *y*_*N*_)}, in which *x*_*i*_ is inputting feature vector and *y*_*i*_ is classification label{+1, −1}. Hyperplane is labeled as (*w*·*x*) + *b* = 0. The optimal hyperplane problem is constructed as follows.


(5) $$\begin{array}{l} \begin{array}{l} \underset{\alpha }{\min}\frac{1}{2}\overset{N}{\underset{i=1}{\sum }}\overset{N}{\underset{j=1}{\sum }}\alpha_{i}\alpha_{j}y_{i}y_{j}\left(x_{i}\cdot x_{j}\right)-\overset{N}{\underset{i=1}{\sum }}\alpha_{i}.\\ s.t.\;\overset{N}{\underset{i=1}{\sum }}\alpha_{i}y_{i}=0,\;\alpha_{i}\geq 0,\;i=1,2,\ldots ,N. \end{array} \end{array}$$

By the Lagrange optimization method, the optimal solution $\alpha^{*}={\left(\alpha_{1}^{*},\;\alpha_{2}^{*},\ldots ,\;\alpha_{N}^{*}\right)}^{T}$ is obtained. The optimal classification function can be given as follows.


(6) $$\begin{array}{l} f\left(x\right)=\text{sgn}\left\{\overset{N}{\underset{i=1}{\sum }}\alpha_{i}^{*}y_{i}\left(x_{i}\cdot x\right)+b^{*}\right\}. \end{array}$$

Where b^*^ is a classification threshold.

For the linearly separable dataset, linear SVM is suitable. However, for a nonlinear dataset, in order to solve the linear inseparable problem, the kernel function could be utilized to map the characteristics of nonlinear separable data points from a relatively low dimension to a relatively high dimension and calculate the relationship between them. The algorithm process of searching the optimal classification hyperplane in the high-dimensional feature space is similar to linear separable SVM, which utilizes kernel function to replace the point product in the high-dimensional feature space. The common kernel functions contain linear kernel, polynomial kernel, radial basis function (rbf), and Sigmoid kernel function, which are defined as followed.


(7) $$\begin{array}{l} K_{linear}\left(x_{i},x_{j}\right)=x_{i}\cdot x_{j}. \end{array}$$

(8) $$\begin{array}{l} K_{polynomial}\left(x_{i},x_{j}\right)={\left(\left(x_{i}\cdot x_{j}\right)+1\right)}^{d}. \end{array}$$

(9) $$\begin{array}{l} K_{rbf}\left(x_{i},x_{j}\right)=\exp\left(-\frac{{\left||x_{i}-x_{j}|\right|}^{2}}{2\sigma^{2}}\right). \end{array}$$

(10) $$\begin{array}{l} K_{sigmoid}\left(x_{i},x_{j}\right)=\tanh\left(k\left(x_{i}\cdot x_{j}\right)+\theta \right). \end{array}$$

Where *d* is an order of polynomial, σ is the radius of radial basis, *k* is a scalar and θ is a shifting value.

### forgeNet_SVM

In order to improve the classification accuracy of SVM, especially for high-dimensional datasets, a new classifier based on forgeNet and SVM (forgeNet_SVM) is proposed in this paper. ForgeNet can not only be utilized for classification but also score the features in the dataset to indicate the importance of the features. Therefore, in forgeNet_SVM algorithm, for high-dimensional datasets, the forgeNet algorithm is used to select important features for feature extraction. In the next step, the important features are input into SVM for learning to solve the classification problem.

### Alzheimer-Related Active Compound Identification

**Figure 2:**
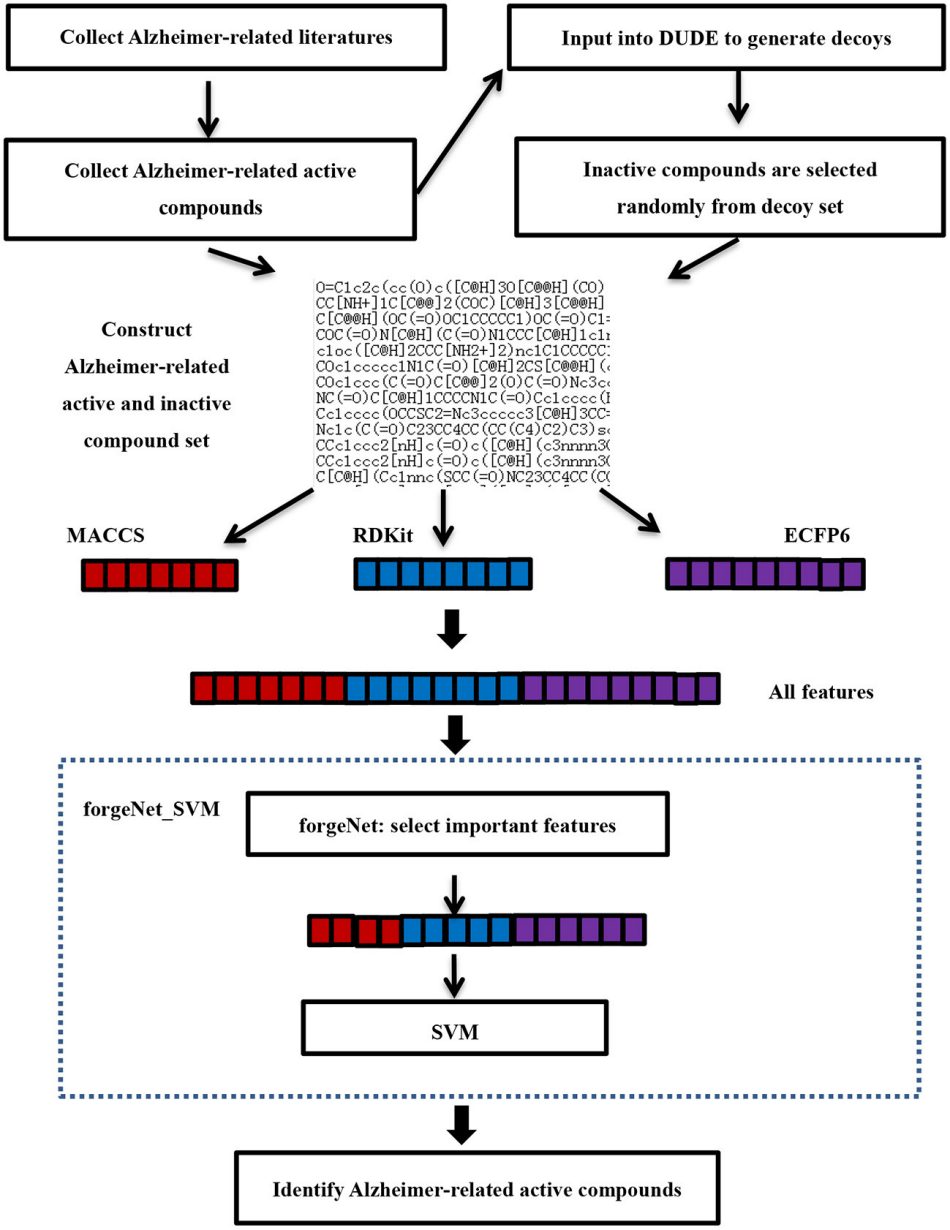
Flowchart of Alzheimer-related active compound identification by forgeNet_SVM.

Figure 2 is the flowchart of Alzheimer-related active compound identification by forgeNet_SVM. The detailed algorithm is given as follows.

1. Studies on TCM in the treatment of AD have to be searched in the literature databases. The queried works of literature need to be analyzed and then collected and mined for important drugs and prescriptions for the treatment of AD, which contains Epimedii Folium, *Anemarrhena asphodeloides*, Radix Ginseng-Poria drug pair, Bajitian, and Polygni Multiflori Caulis. Next, *m*Alzheimer-related closely active compounds, such as naringin, quercetin, Kaempferol, β-Sitosterol, Isorhamnetin, Stigmasterol, and Icariin have to be retrieved. These important compounds have been verified by biological experiments or the molecular docking method. *m* active compounds are utilized as positive samples for further data analysis. In order to determine the negative sample, *m* active compounds are input to the UDU-E website to generate the corresponding decoys (Mysinger et al., 2012). In order to set up the inactive compound set (negative samples), the random decoy selection is performed 3 *m* times from the obtained decoy sets without putting it back. Thus, the inactive compound set contains 3 *m* compounds. The sets of active and inactive compounds constitute the compound sample dataset.
2. The molecular structures of compounds in the dataset collected are SMILES (simplified molecular input line entry system). According to the SMILES structures, three molecular descriptors (ECFP6, MACCS, and RDKit) are utilized to obtain the feature sets of compounds respectively. ECFP6 (*e*_1_, *e*_2_, …, *e*_*n*_*e*__), MACCS (*m*_1_, *m*_2_, …, *m*_*n*_*m*__) and RDKit (*r*_1_, *r*_2_, …, *r*_*n*_*r*__) feature sets of each compound are fused into an all_feature set (*e*_1_, *e*_2_, …, *e*_*n*_*e*__, *m*_1_, *m*_2_, …, *m*_*n*_*m*__, *r*_1_, *r*_2_, …, *r*_*n*_*r*__), where *n*_*e*_, *n*_*m*_, and *n*_*r*_are the numbers of ECFP6, MACCS, and RDKit feature sets, respectively. The forgeNet_SVM is utilized to identify Alzheimer-related compounds according to the dataset collected. In order to improve the classification performance of the classifier, all features are input to the forgeNet, which could be utilized to provide the importance of each feature. According to the score of each feature, the important features for classification are selected in order to achieve the purpose of feature extraction. The selected feature set is give as [*d*_1_, *d*_2_, …, *d*_*n*_].Next, the selected features are input to SVM algorithm for learning. The features of new compounds in TCM prescription are extracted with the same method, which are input to SVM in order to be identified.

## Experiments and Discussions

In order to test the effectiveness of the proposed method in this paper, the prescriptions and drugs for treating AD are searched. In total 94 Alzheimer-related active compounds are collected and 282 unrelated compounds are also obtained. Each compound is extracted by ECFP6, MACCS, and RDKit to obtain three feature sets (ECFP6, MACCS, and RDKit), respectively. These three feature sets are combined, and a total of 2,423 features are obtained for each compound as the all_feature set. In order to evaluate the performance of the method, TPR, FPR, Precision, Specificity, F1, ROC, and AUC are applied. Seven classical classifiers containing AdaBoost (Cao et al., 2013), Gradient Boosting Decision Tree (GBDT) (Hu and Min, 2018), K-Nearest Neighbor (KNN) (Denoeux, 1995), logistic regression (LR) (Maalouf, 2011), naive Bayes (NB) (Rish, 2001), random forest (RF) (Breiman, 2001), and decision tree (DT) (Breiman et al., 1984)) are also utilized to identify the compounds about Alzheimer. In forgeNet_SVM, the number of trees is set to 1,000, random forest is utilized, three hidden layers are contained, the learning rate is set as 0.0001, the number of training epochs is set to 50, and the linear kernel is selected as the kernel function. In GBDT, the maximum number of weak learners is set to 200. In LR, *L*2 norm is utilized to constrain the arguments. In RF, the number of decision trees is set to 100, the bootstrap method is utilized and the number of features is set to $\sqrt{n\text{\_}features}$(*n*_*features* is the number of features) when searching for the best segmentation.

**Figure 3:**
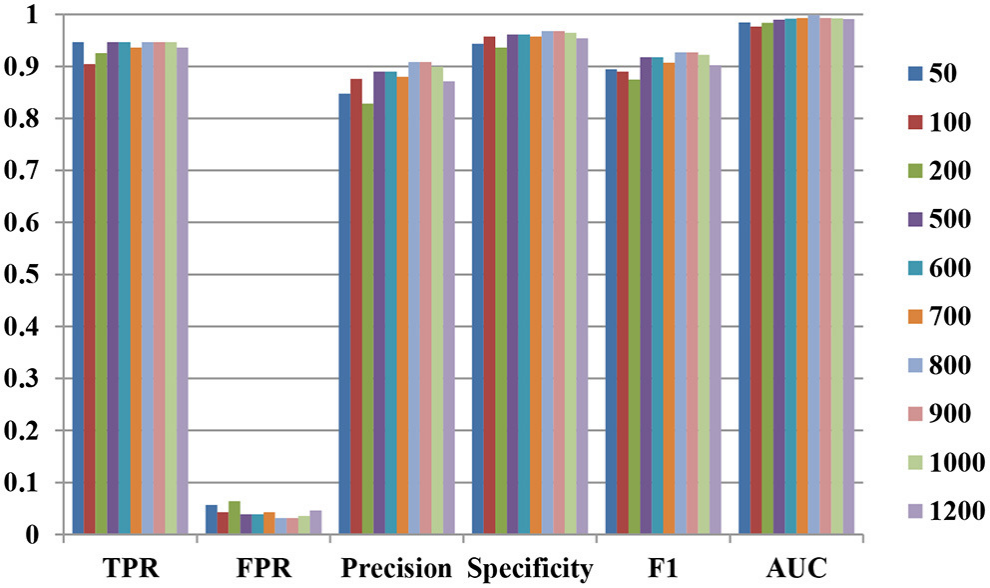
Performances of forgeNet_SVM with the different numbers of features.

For forgeNet_SVM, forgeNet can select the important features from a large number of feature sets. First, the different numbers of features are tested for affecting the performance of our method. The numbers of important features selected by forgeNet are 50, 100, 200, 500, 600, 700, 800, 900, 1,000, and 1,200. With the different numbers of feature sets, by 10-cross validation method, the performances of TPR, FPR, Precision, Specificity, *F1*, ROC, and AUC obtained are shown in Figure 3. The 10-cross validation method is utilized to divide the training and testing datasets in order to evaluate the model. From Figure 3, we can see that our method performs best in terms of TPR when selecting 50, 500, 600, 800, 900, and 1,000 features. In terms of FPR, Precision, Specificity, and F1, our method performs best when selecting 800 and 900 features. Through the results, we can see that our method performs best when 800 and 900 features are selected. In the following experiment, we select the first 900 important features as feature set by forgeNet.

**Figure 4:**
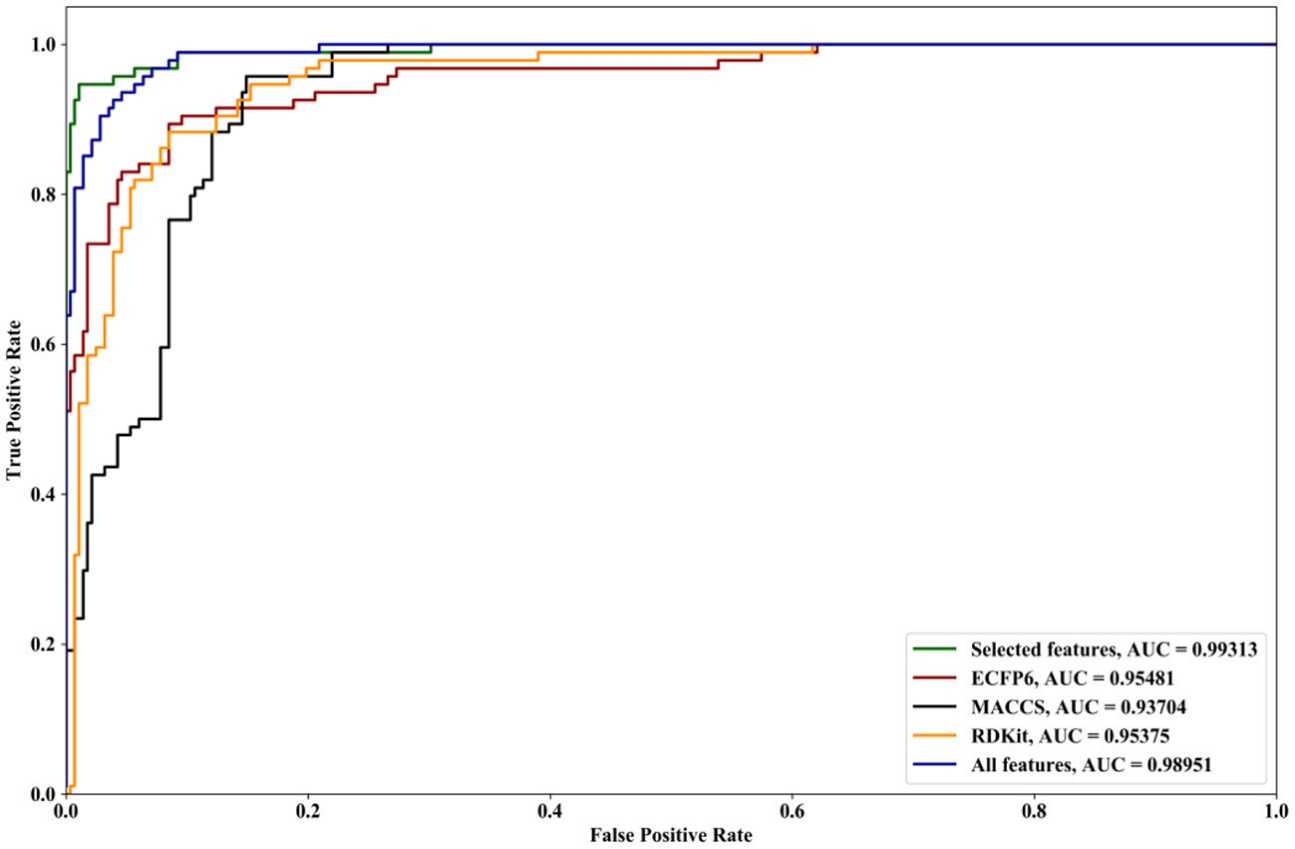
ROC curves and AUC performances of our method with different feature sets for Alzheimer-related compound identification with Dat1.

**Figure 5:**
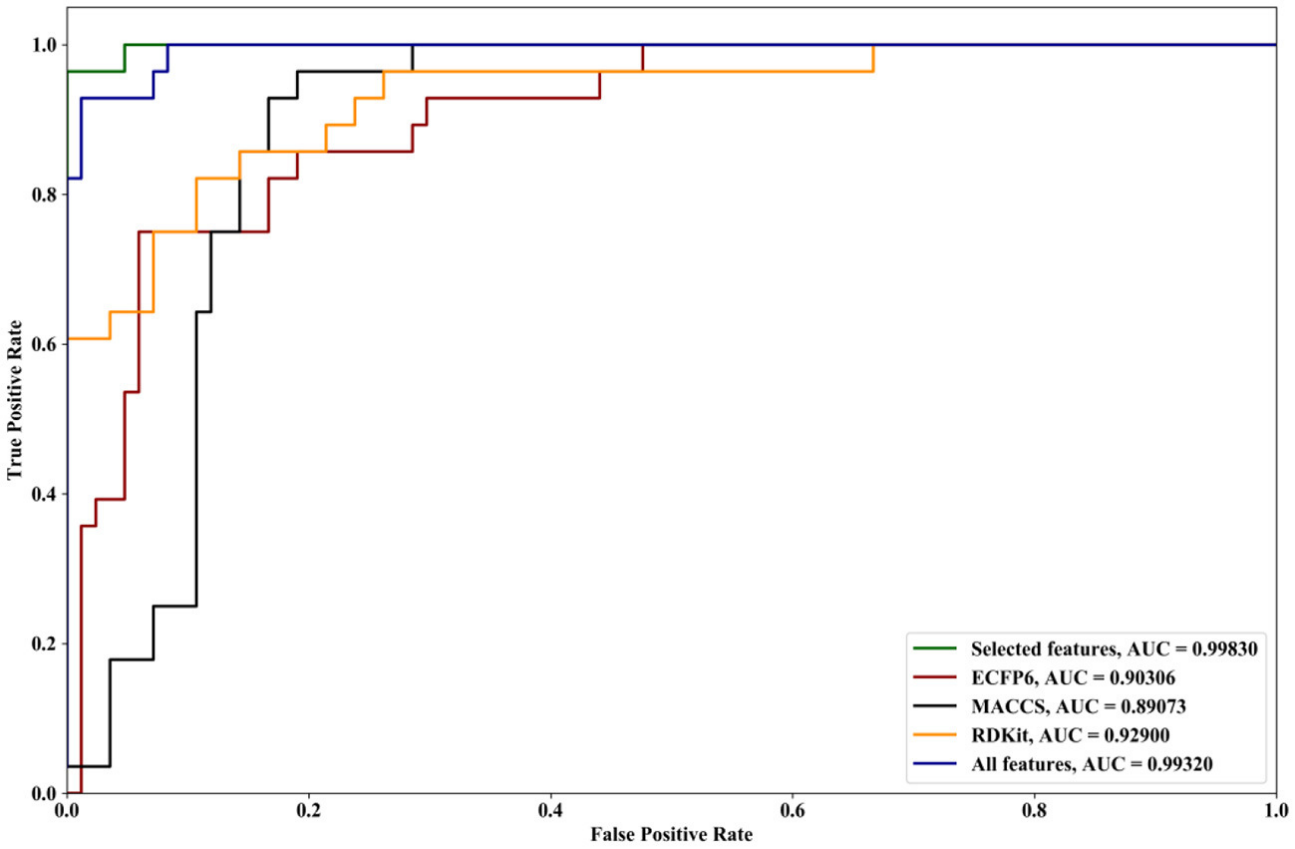
ROC curves and AUC performances of our method with different feature sets for Alzheimer-related compound identification with Dat2.

We compare the effects of different feature sets on the performance of the algorithm. The feature sets include ECFP6, MACCS, and RDKit, and all features and selected features are obtained by forgeNet. Two datasets are utilized. The first dataset contains all the compounds (**Dat1**), and another one is obtained by random division (**Dat2**) in which 70% of compounds are used as the training set and the remaining compounds are as the testing set. With Dat1, using the 10-cross validation method, the performances of our method with different feature sets for Alzheimer-related compound identification are shown in Figure 4 and Table 1. From Figure 4, it could be seen that the selected feature set has better ROC curves than three single feature sets (ECFP6, MACCS, and RDKit) and all features. Furthermore, in terms of AUC, the selected feature set is 4% higher than ECFP6, 6% higher than MACCS, 4.1% higher than RDKit, and 0.4% higher than the all_feature set. From Table 1, it could be seen that in terms of TPR, FPR, Precision, Specificity, and F1, the selected feature set performs better than ECFP6, MACCS, RDKit, and the all_feature sets. With Dat2 and the different feature sets, the identification results of active compounds are shown in Figure 5 and Table 2. From Figure 5, the selected features are utilized to obtain a better ROC curve than the other four feature sets. In terms of AUC, the selected feature set is 4, 6, 4.1, and 0.37% higher than ECFP6, MACCS, RDKit, and the all_feature sets, respectively. Table 2 shows that our selected features could make SVM obtain the best performances of TPR, FPR, Precision, Specificity, and F1. From all the results, it could be seen that the merged feature set (all features) performs better than the three single feature sets (ECFP6, MACCS, and RDKit). Using the forgeNet, the important features could be selected, so the selected feature set could obtain better performances than the merged feature set in terms of TPR, FPR, Precision, Specificity, and F1. Thus the feature extraction method can improve the accuracy of active compound recognition.

**Table 1:** Performances of our method with different feature sets for Alzheimer-related compound identification with Dat1.

**Feature sets**	**TPR**	**FPR**	**Precision**	**Specificity**	**F1**
Selected features	**0.946809**	**0.031915**	**0.908163**	**0.968085**	**0.927083**
ECFP6	0.829787	0.060284	0.821053	0.939716	0.825397
MACCS	0.882979	0.124113	0.70339	0.875887	0.783019
RDKit	0.882979	0.106383	0.734513	0.893617	0.801932
All features	0.93617	0.056738	0.846154	0.943262	0.888889

*The bold values denote the best performances*.

**Table 2:** Performances of our method with different feature sets for Alzheimer-related compound identification with Dat2.

**Feature sets**	**TPR**	**FPR**	**Precision**	**Specificity**	**F1**
Selected features	**0.964286**	**0**	**1**	**1**	**0.981818**
ECFP6	0.678571	0.059524	0.791667	0.940476	0.730769
MACCS	0.821429	0.142857	0.657143	0.857143	0.730159
RDKit	0.857143	0.214286	0.571429	0.785714	0.685714
All features	0.678571	0.059524	0.791667	0.940476	0.730769

*The bold values denote the best performances*.

AdaBoost, GBDT, KNN, LR, NB, RF, and DT are also directly utilized to predict Alzheimer-related compounds with Dat1 and Dat2. In forgeNet_SVM, SVM is also replaced with these seven classifiers in order to constitute forgeNet_AdaBoost, forgeNet_GBDT, forgeNet_KNN, forgeNet_LR, forgeNet_NB, forgeNet_RF, and forgeNet_DT, which are utilized to identify compounds. With Dat1 and Dat2, the performances of 15 methods for Alzheimer-related compound identification are listed in Tables 3, 4, respectively. From Table 3, KNN and LR could obtain the best TPR performance, which shows that KNN and LR could identify the most active compounds. But these two methods shave the worst FPR performances, which are 0.77305 and 0.56383, respectively. The results reveal that LR identifies most of the compounds as active compounds. In terms of FPR, Precision, and Specificity, NB performs best. But NB has the worst TPR performance, which shows that NB identifies most of the compounds as inactive compounds. In terms of F1 and AUC, forgeNet_SVM could obtain the best performances among the 15 methods. From Table 4, KNN and LR could gain the best TPR performance, which reveals that these two methods could identify all true active compounds. forgeNet_SVM, forgeNet_NB, and forgeNet_DT could obtain the second better TPR performance. ForgeNet_SVM could gain the best FPR performance, which shows that our proposed method can identify all true inactive compounds. In terms of Precision, Specificity, F1, and AUC, forgeNet_SVM also performs best. On the whole, our proposed method could infer more true active and inactive compounds than other methods.

**Table 3:** Performances of 15 methods for Alzheimer-related compound identification with Dat1.

**Methods**	**TPR**	**FPR**	**Precision**	**Specificity**	**F1**	**AUC**
forgeNet_SVM	0.946809	0.031915	0.908163	0.968085	**0.927083**	**0.99313**
AdaBoost	0.914894	0.035461	0.895833	0.964539	0.905263	0.974083
forgeNet_AdaBoost	0.914894	0.035461	0.895833	0.964539	0.905263	0.974083
GBDT	0.904255	0.039007	0.885417	0.960993	0.894737	0.981326
forgeNet_GBDT	0.914894	0.028369	0.914894	0.971631	0.914894	0.982383
KNN	**0.989362**	0.77305	0.299035	0.22695	0.459259	0.798759
forgeNet_KNN	0.893617	0.028369	0.913043	0.971631	0.903226	0.978101
LR	**0.989362**	0.56383	0.369048	0.43617	0.537572	0.942282
forgeNet_LR	0.93617	0.042553	0.88	0.957447	0.907216	0.942282
NB	0.287234	**0**	**1**	**1**	0.446281	0.643617
forgeNet_NB	0.946809	0.039007	0.89	0.960993	0.917526	0.962464
RF	0.882979	0.031915	0.902174	0.968085	0.892473	0.98823
forgeNet_RF	0.904255	0.031915	0.904255	0.968085	0.904255	0.986457
DT	0.87234	0.109929	0.725664	0.890071	0.792271	0.881206
forgeNet_DT	0.946809	0.060284	0.839623	0.939716	0.89	0.943262

*The bold values denote the best performances*.

**Table 4:** Performances of 15 methods for Alzheimer-related compound identification with Dat2.

**Methods**	**TPR**	**FPR**	**Precision**	**Specificity**	**F1**	**AUC**
forgeNet_SVM	0.964286	**0**	**1**	**1**	**0.981818**	**0.998299**
AdaBoost	0.357143	0.309524	0.277778	0.690476	0.3125	0.991071
forgeNet_AdaBoost	0.892857	**0**	**1**	**1**	0.943396	0.995748
GBDT	0.821429	0.607143	0.310811	0.392857	0.45098	0.997449
forgeNet_GBDT	0.928571	**0**	**1**	**1**	0.962963	0.993197
KNN	**1**	**1**	0.25	0	0.4	0.742347
forgeNet_KNN	0.892857	0.035714	0.892857	0.964286	0.892857	0.94494
LR	**1**	0.678571	0.329412	0.321429	0.495575	0.964711
forgeNet_LR	0.928571	0.071429	0.8125	0.928571	0.866667	0.985544
NB	0	**0**		**1**		0.5
forgeNet_NB	0.964286	0.059524	0.84375	0.940476	0.9	0.951743
RF	0.535714	0.130952	0.576923	0.869048	0.555556	0.987724
forgeNet_RF	0.928571	**0**	**1**	**1**	0.962963	0.996173
DT	0.857143	0.630952	0.311688	0.369048	0.457143	0.839286
forgeNet_DT	0.964286	0.011905	0.964286	0.988095	0.964286	0.97619

*The bold values denote the best performances*.

## Conclusion

In this study, a novel Alzheimer-related compound identification algorithm based on data fusion and forgeNet_SVM is proposed. Three feature description methods (ECFP6, MACCS, and RDKit) are utilized to obtain the feature sets of Alzheimer related and unrelated compounds, which are fused into the all_feature set. In forgeNet_SVM, all_feature set is input to forgeNet, which could evaluate the importance of each feature and extract the important features according to the given scores. The selected features are input to SVM algorithm to identify the new compounds in a TCM prescription. The Alzheimer-related dataset collected is utilized, and the experiment results show that forgeNet_SVM could identify more true-positive compounds and fewer false-positive compounds than other classical classifiers, such as AdaBoost, GBDT, KNN, LR, NB, RF, and DT. We make the comparison experiments that give the optimal number of the selected features for forgeNet_SVM. In terms of TPR, FPR, Precision, Specificity, F1, and AUC, the selected feature set performs better than the all_feature set and three single feature sets (ECFP6, MACCS, and RDKit).

In the future, we will apply forgeNet_SVM to identify other diseases related compounds, such as cancer, COVID-19, and cardiovascular diseases.

## Data Availability Statement

The original contributions presented in the study are included in the article/supplementary material, further inquiries can be directed to the corresponding authors.

## Author Contributions

WB conceived the method. BY designed the method and conducted the experiments. WB and SH wrote the main manuscript text. All authors reviewed the manuscript.

## Funding

This work was supported by the Talent Project of Qingtan Scholar of Zaozhuang University, the Natural Science Foundation of China (No. 61902337), the Fundamental Research Funds for the Central Universities (2020QN89), Xuzhou Science and Technology Plan Project (KC19142 and KC21047), Shandong Provincial Natural Science Foundation, China (No. ZR2015PF007), Jiangsu Provincial Natural Science Foundation (No. SBK2019040953), Natural Science Fund for Colleges and Universities in Jiangsu Province (No. 19KJB520016), and Young Talents of Science and Technology in Jiangsu.

## Conflict of Interest

The authors declare that the research was conducted in the absence of any commercial or financial relationships that could be construed as a potential conflict of interest.

## Publisher's Note

All claims expressed in this article are solely those of the authors and do not necessarily represent those of their affiliated organizations, or those of the publisher, the editors and the reviewers. Any product that may be evaluated in this article, or claim that may be made by its manufacturer, is not guaranteed or endorsed by the publisher.
