# Corrigendum: Alzheimer-compound identification based on data fusion and forgeNet_SVM

**DOI:** 10.3389/fnagi.2023.1322944

**Published:** 2023-11-17

**Authors:** Bin Yang, Wenzheng Bao, Shichai Hong

**Affiliations:** ^1^School of Information Science and Engineering, Zaozhuang University, Zaozhuang, China; ^2^School of Information and Electrical Engineering, Xuzhou University of Technology, Xuzhou, China; ^3^Department of Vascular Surgery, Zhongshan Hospital (Xiamen), Fudan University, Xiamen, China

**Keywords:** virtual screening, network pharmacology, Alzheimer, data fusion, feature selection, machine learning

In the published article, there was an error in affiliation 3. Instead of “[Department of Vascular Surgery, Zhongshan Hospital, Fudan University, Xiamen, China”, it should be “Department of Vascular Surgery, Zhongshan Hospital (Xiamen), Fudan University, Xiamen, China”.

The authors apologize for this error and state that this does not change the scientific conclusions of the article in any way. The original article has been updated.

